# Hemostatic matrix for treating intracollection bleeding after endoscopic ultrasound-guided drainage and endoscopic necrosectomy of walled-off pancreatic necrosis in a patient with cirrhosis

**DOI:** 10.1055/a-2085-0109

**Published:** 2023-06-12

**Authors:** Giacomo Emanuele Maria Rizzo, Dario Ligresti, Lucio Carrozza, Salvatore Tammaro, Mario Traina, Ilaria Tarantino

**Affiliations:** 1 Endoscopy Service, Department of Diagnostic and Therapeutic Services, IRCCS – ISMETT, Palermo, Italy; 2 Department of Surgical, Oncological and Oral Sciences (Di.Chir.On.S.), University of Palermo, Palermo, Italy

A 45-year-old man with alcoholic liver cirrhosis was referred to our institute for treatment of infected walled-off pancreatic necrosis (WOPN) 6 months after an episode of acute pancreatitis. Computed tomography confirmed cirrhotic liver disease with collateral vessels and a pancreatic fluid collection at the body-tail (9 × 3 cm). Laboratory tests showed low platelets (55 000/mm ^3^ ) and international normalized ratio of 1.5. Endoscopic ultrasound (EUS) evaluation showed perigastric collateral circulation and a WOPN with necrotic material accounting for 90 % of the total volume ( Fig. 1 ).

**Fig. 1:**
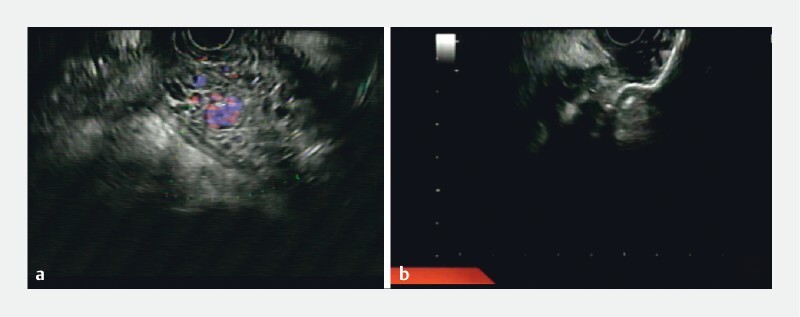
Endoscopic ultrasound view of the walled-off pancreatic necrosis (WOPN). **a** Before deployment of the lumen-apposing metal stent (LAMS), color Doppler showed a tight relationship between collateral vessels into the cavity and the WOPN. **b** During LAMS deployment.

We performed EUS-guided drainage of the collection by deploying a lumen-apposing metal stent (LAMS; type HOT-AXIOS, 10 mm length, 20 mm diameter; Boston Scientific, Marlborough, Massachusetts, USA) with creation of a cysto-gastrostomy, which was dilated to 20 mm using a balloon. During the same session, we performed necrosectomy using forceps and endoscopic snare, during which spurting bleeding suddenly occurred from a vessel in the wall ( Fig. 2 ); hemostasis was achieved immediately by using a metal clip.

**Fig. 2:**
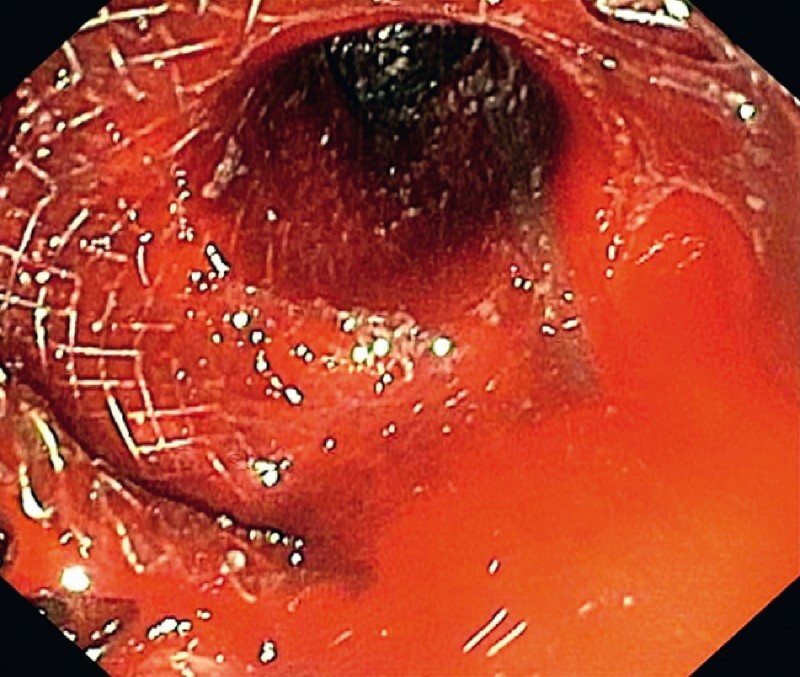
Endoscopic view of bleeding during the first necrosectomy session.

The patient was closely monitored until he underwent further necrosectomy sessions, with gradual improvement in clinical, laboratory, and endoscopic status. At the fourth necrosectomy session, we cleaned the cavity by removing the residual necrotic material and observed granulation tissue on the wall; however, a wide area of the collection showed oozing bleeding, so we decided to apply a hemostatic agent (PuraStat; 3-D Matrix Medical Technology, Tokyo, Japan) into the cavity, next to the damaged wall, which immediately stopped the bleeding ( Fig. 3 , Video 1 ). We kept the LAMS in place for a further 48 hours in case of rebleeding, and after an endoscopy to check the area, we removed it.

**Fig. 3:**
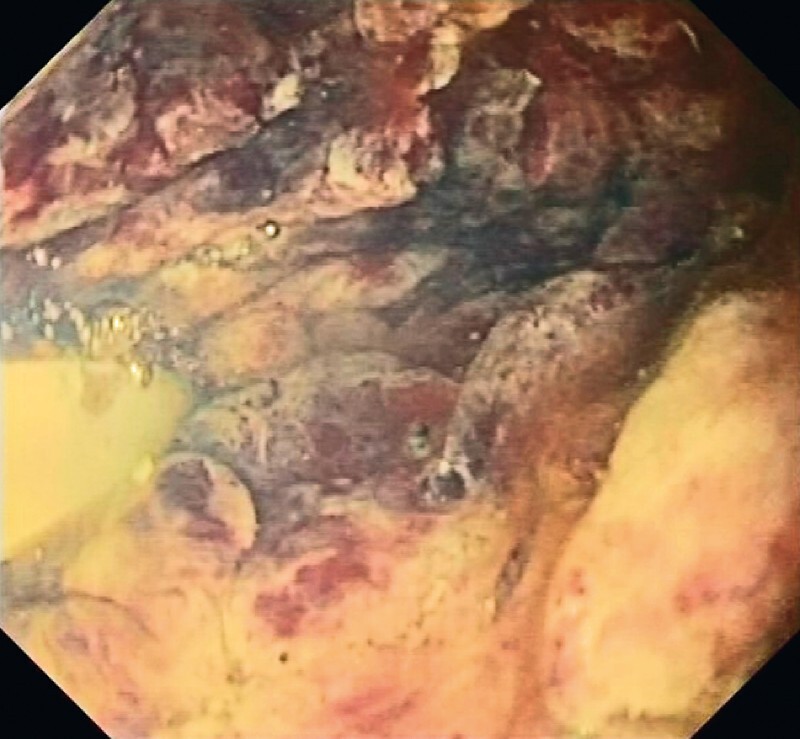
Endoscopic view during the last necrosectomy session while deploying the hemostatic matrix. No more bleeding was seen from the wall of the collection.

**Video 1** Endoscopic ultrasound-guided drainage of walled-off pancreatic necrosis, with management of intracavity bleeding using a hemostatic matrix, in a patient with cirrhosis.


The patient’s clinical and laboratory indices improved, and he was discharged 24 hours after LAMS removal. After 2 months of follow-up he had no complaints or symptoms. To our knowledge, this is the first case report of hemostatic matrix application as a single agent for treatment of intracollection bleeding following necrosectomy in a patient with liver cirrhosis.

Endoscopy_UCTN_Code_CPL_1AK_2AG

## Notes

Endoscopy E-Videos

https://eref.thieme.de/e-videos
*E-Videos* is an open access online section of the journal *Endoscopy* , reporting on interesting cases and new techniques in gastroenterological endoscopy. All papers include a high-quality video and are published with a Creative Commons CC-BY license. Endoscopy E-Videos qualify for HINARI discounts and waivers and eligibility is automatically checked during the submission process. We grant 100% waivers to articles whose corresponding authors are based in Group A countries and 50% waivers to those who are based in Group B countries as classified by Research4Life (see: https://www.research4life.org/access/eligibility/ ). This section has its own submission website at https://mc.manuscriptcentral.com/e-videos

